# Anharmonicity Reveals the Tunability of the Charge Density Wave Orders in Monolayer VSe_2_

**DOI:** 10.1021/acs.nanolett.2c04584

**Published:** 2023-02-24

**Authors:** Adolfo Otero Fumega, Josu Diego, Victor Pardo, Santiago Blanco-Canosa, Ion Errea

**Affiliations:** † Department of Applied Physics, Aalto University, 02150 Espoo, Finland; ‡ Fisika Aplikatua Saila, Gipuzkoako Ingeniaritza Eskola, University of the Basque Country (UPV/EHU), 20018 San Sebastián, Spain; ¶ Centro de Física de Materiales (CSIC-UPV/EHU), 20018 San Sebastián, Spain; § Departamento de Física Aplicada, Universidade de Santiago de Compostela, 15782 Santiago de Compostela, Spain; ∥ Instituto de Materiais iMATUS, Universidade de Santiago de Compostela, 15782 Santiago de Compostela, Spain; ⊥ Donostia International Physics Center (DIPC), 20018 San Sebastián, Spain; # IKERBASQUE, Basque Foundation for Science, 48013 Bilbao, Spain

**Keywords:** anharmonic effects, charge density wave, competing orders, 2D materials, van der Waals interactions, strain

## Abstract

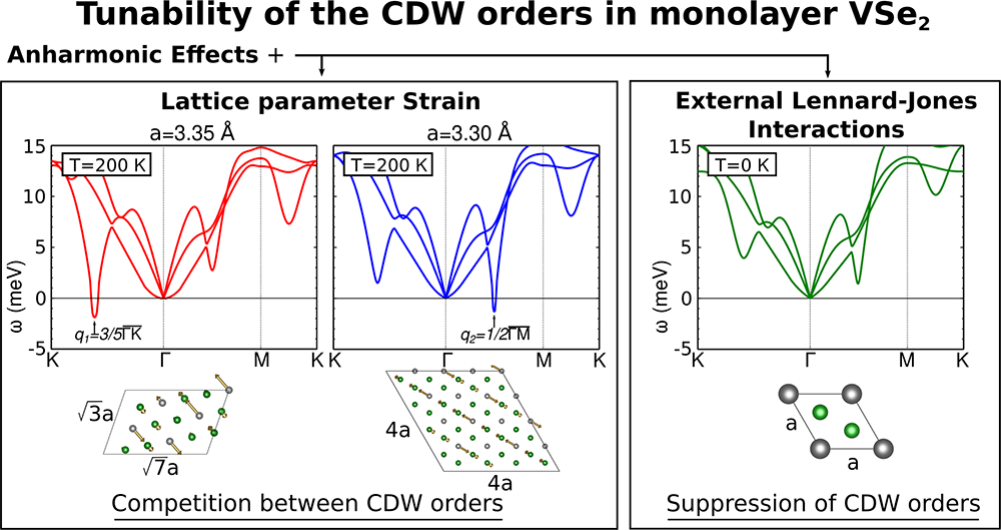

VSe_2_ is a layered compound that has attracted great attention due to its proximity to a ferromagnetic state that is quenched by its charge density wave (CDW) phase. In the monolayer limit, unrelated experiments have reported different CDW orders with different transition temperatures, making this monolayer very controversial. Here we perform first-principles nonperturbative anharmonic phonon calculations in monolayer VSe_2_ in order to estimate the CDW order and the corresponding transition temperature. They reveal that monolayer VSe_2_ develops two independent charge density wave orders that compete as a function of strain. Variations of only 1.5% in the lattice parameter are enough to stabilize one order or the other. Moreover, we analyze the impact of external Lennard-Jones interactions, showing that these can act together with anharmonicity to suppress the CDW orders. Our results solve previous experimental contradictions, highlighting the high tunability and substrate dependency of the CDW orders of monolayer VSe_2_.

Two-dimensional (2D) materials are an ideal platform to artificially engineer heterostructures with new functionalities due to the weak van der Waals bonding between layers.^1^ Monolayers hosting symmetry-broken phases, such as superconductivity,^2,3^ magnetism,^4−8^ ferroelectricity,^9,10^ charge density waves (CDWs),^11,12^ or multiferroicity,^13,14^ represent the most interesting building blocks to design novel phases of matter. One of the main challenges in the task of engineering novel functional materials with broken-symmetry monolayers is to overcome the restrictions imposed by the reduced dimensionality,^15,16^ which may prevent the formation of these phases, and the competition between ordered phases due to the subtle interplay of different interactions.^17,18^ For instance, CDW phases have been reported to destroy^19,20^ or promote^21^ 2D ferromagnetism. VSe_2_ is a paradigmatic example of this as, despite some early claims,^22^ it is now clear both experimentally and theoretically that the CDW order quenches the emergence of itinerant ferromagnetism.^19,20,23−28^ In its bulk form, VSe_2_ develops a commensurate 4 × 4 × 3 CDW phase below 110 K.^29^ The CDW phase opens pseudogaps at the Fermi level impeding the emergence of ferromagnetism.^19^ Inelastic X-ray scattering experiments and nonperturbative anharmonic phonon calculations have proven that the CDW transition is driven by the collapse of a low-energy acoustic mode and that the electron–phonon coupling is the origin of the instability,^30^ as suggested as well by other quantitative models.^31^ These anharmonic calculations have shown that van der Waals interactions are essential to melt the CDW and obtain a charge density wave temperature (*T*_CDW_) in agreement with experiments. This suggests that the CDW in the monolayer may also be characterized by similar phonon softening effects but with limited influence of van der Waals interactions.

The main problem in the monolayer of VSe_2_ is that the CDW is not fully understood yet, as unrelated experiments have reported distinct CDW orders with different transition temperatures. A nonmonotonic evolution of *T*_CDW_ as a function of the number of layers has been reported in refs (32)–^34^ but retaining an in-plane 4 × 4 modulation. A metastable phase with modulation  has also been identified for the few-layer case.^35^ In the purely 2D limit different CDW orders with nonequivalent modulations have been found. A 4 × 4 order was observed in VSe_2_ films grown on bilayer graphene on top of SiC and on highly oriented pyrolytic graphite (HOPG) with a *T*_CDW_ of  K and a lattice parameter of *a* = 3.31 ± 0.05 Å.^27^ On the contrary, a  modulation has been observed in VSe_2_ samples grown on several substrates by molecular beam epitaxy by different groups, with a consistent *T*_CDW_ = 220 K.^20,23^ Some other orders have also been reported: a combination of  and  with a *T*_CDW_ ∼ 135 K^25,26^ and a 4 × 1 modulation with *T*_CDW_ ∼ 350 K.^26,36^ These experimental contradictions point to the presence of different competing CDW orders, which can lead to different low-temperature phases depending on the substrate.^26,36^

By calculating the harmonic phonons of the VSe_2_ monolayer within density functional theory (DFT), theoretical studies have also described the competition of different CDW orders and how strain can influence the ground state.^37^ Harmonic phonon calculations, however, cannot explain that above *T*_CDW_ the 1T phase is the ground state. In the presence of competing orders, only calculations considering anharmonicity can disentangle what is the CDW order and the transition temperature, as it has already been shown in different transition metal dichalcogenides (TMDs).^30,38−41^ Therefore, in order to unveil the intrinsic CDW orders of monolayer VSe_2_ and how they are affected by external fields, a DFT study including anharmonicity is required.

In this work, we present a theoretical analysis of the CDW orders arising in monolayer VSe_2_ using nonperturbative anharmonic phonon calculations based on the stochastic self-consistent harmonic approximation (SSCHA).^42−45^ This formalism has been crucial to understand and characterize the CDWs in several TMDs^30,38−41^ as it overcomes the limitations of the harmonic analysis, allowing to determine the dependence of the CDW order as a function of temperature. We demonstrate the emergence and competition of two intrinsic CDW orders in monolayer VSe_2_ as for a lattice parameter of *a* = 3.35 Å a  order dominates with *T*_CDW_ = 217 K, while for slightly smaller *a* = 3.30 Å the 4 × 4 order prevails with *T*_CDW_ = 223 K. Moreover, the nonperturbative anharmonic procedure allows us to demonstrate that the CDW can be suppressed by the inclusion of Lennard-Jones energy terms, which might appear naturally or may be artificially induced by the interplay between the monolayer and a particular substrate.

We start our analysis performing harmonic phonon calculations on monolayer VSe_2_. The normal state (NS) unit cell is shown in Figure 1a. The value of the experimentally reported lattice parameter *a* = 3.31 ± 0.05 Å is in rather good agreement with the theoretical one of 3.35 Å obtained at the Perdew–Burke–Ernzerhof^46^ level without considering the zero-point motion.^23,27^ Therefore, in this study we perform calculations for two lattice parameters *a* = 3.35 Å and *a* = 3.30 Å, which provide a good representation of the experimental range. Density functional perturbation theory (DFPT)^47^ is used to compute the harmonic phonon band structure for both lattice parameters (see the Supporting Information for a detailed description of the calculations). Both harmonic phonon bands (see Figure 1b) show two dominant instabilities at  and . These are associated with the two intrinsic CDW orders of monolayer VSe_2_, with modulations shown in Figure 1c,d, that lower its Born–Oppenheimer energy. The instability at *q*_1_ is associated with a  supercell, while the one at *q*_2_ leads to a 4 × 4 modulation. Both softened phonon modes have an out-of-plane component in the displacement vectors, as it is also the case of the CDW instability in the bulk form of this compound. In spite of providing the two intrinsic CDW orders, harmonic calculations do not suffice to predict which of these CDW orders is the dominant one or the associated transition temperature for each lattice parameter. In fact, the small change in the lattice parameter does not impact the weight of the instabilities. Our nonperturbative anharmonic calculations based on a free energy formalism within the SSCHA can give the answer to these questions (see the Supporting Information for a detailed description of the SSCHA method and the technical aspects of these calculations).

**Figure 1:**
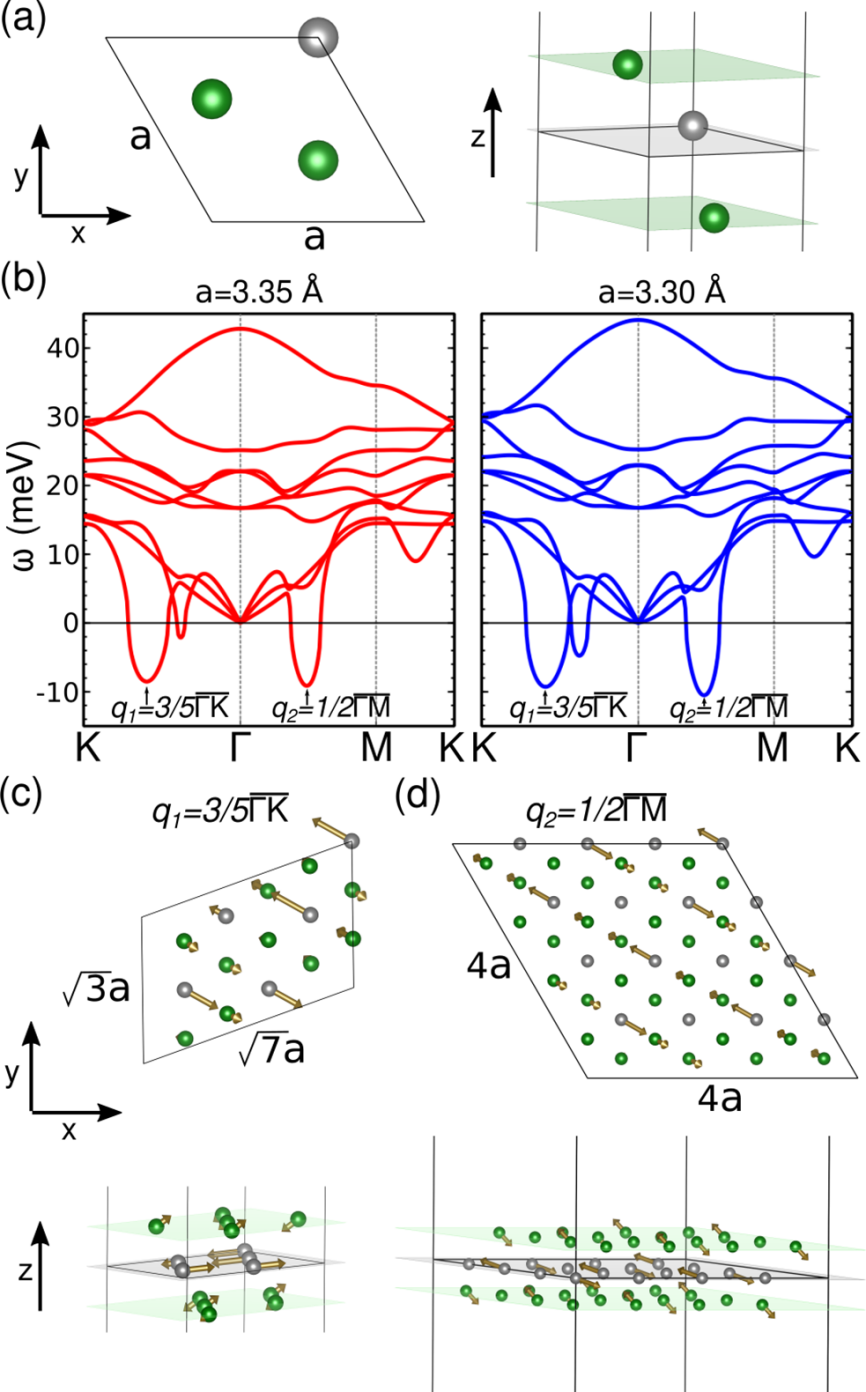
(a) Normal-state structure of monolayer VSe_2_ with lattice parameter *a*. V (Se) atoms are depicted in gray (green). (b) Harmonic phonon band structures of monolayer VSe_2_ as a function of the lattice parameter, left (right) panel for *a* = 3.35 Å (*a* = 3.30 Å). Two dominant instabilities at  and  can be identified. (c,d) Intrinsic CDW orders with  and 4 × 4 modulations associated with the instabilities at *q*_1_ and *q*_2_ can be identified in the harmonic phonon band structures. The displacement vectors associated with each CDW order are plotted as brown arrows. Planes perpendicular to the *z*-direction for V (Se) were plotted in gray (green) for a better characterization of the displacement vectors.

Figure 2 shows the temperature evolution of the phonon band structure obtained with the SSCHA method for the two lattice parameters *a* = 3.35 Å (in red in the top panels of Figure 2) and *a* = 3.30 Å (in blue in the lower panels of Figure 2). At high enough temperature (*T* = 250 K) the 1T NS phase (Figure 1a) is dynamically stable for both lattice parameters as shown in Figures 2a,d, showing that anharmonicity melts the CDW phase as it happens in other TMDs.^30,38−41^ By decreasing the temperature, the phonon modes associated with the CDW instabilities at *q*_1_ and *q*_2_ soften. In particular, for *a* = 3.35 Å at 200 K (Figure 2b) we can observe that the mode at  becomes unstable, even if the one at *q*_2_ remains stable. This result indicates that for this lattice parameter the  CDW order dominates. However, for *a* = 3.30 Å at 200 K (Figure 2e) the phonon mode at  is unstable, but not at *q*_1_. Therefore, for the smaller lattice parameter, the 4 × 4 CDW order is the dominant one. At low enough temperatures (Figures 2c,f) both *q*-vectors show unstable modes. However, note that this situation is not indicating that at low temperatures both CDW orders coexist, although it is a clear signature that the anharmonic free energy landscape becomes more complex. Once one of the CDW orders becomes stable when the temperature is decreased, the system collapses to it, and the analysis in terms of the anharmonic phonons of the NS phase is no longer useful to describe the evolution of each of the CDW phases at low temperatures. Nevertheless, the anharmonic phonons at low temperature shown in Figure 2c,f confirm that both *q*_1_ and *q*_2_ are the intrinsic CDW orders of VSe_2_ that can be accessed through a transition from the NS phase.

**Figure 2:**
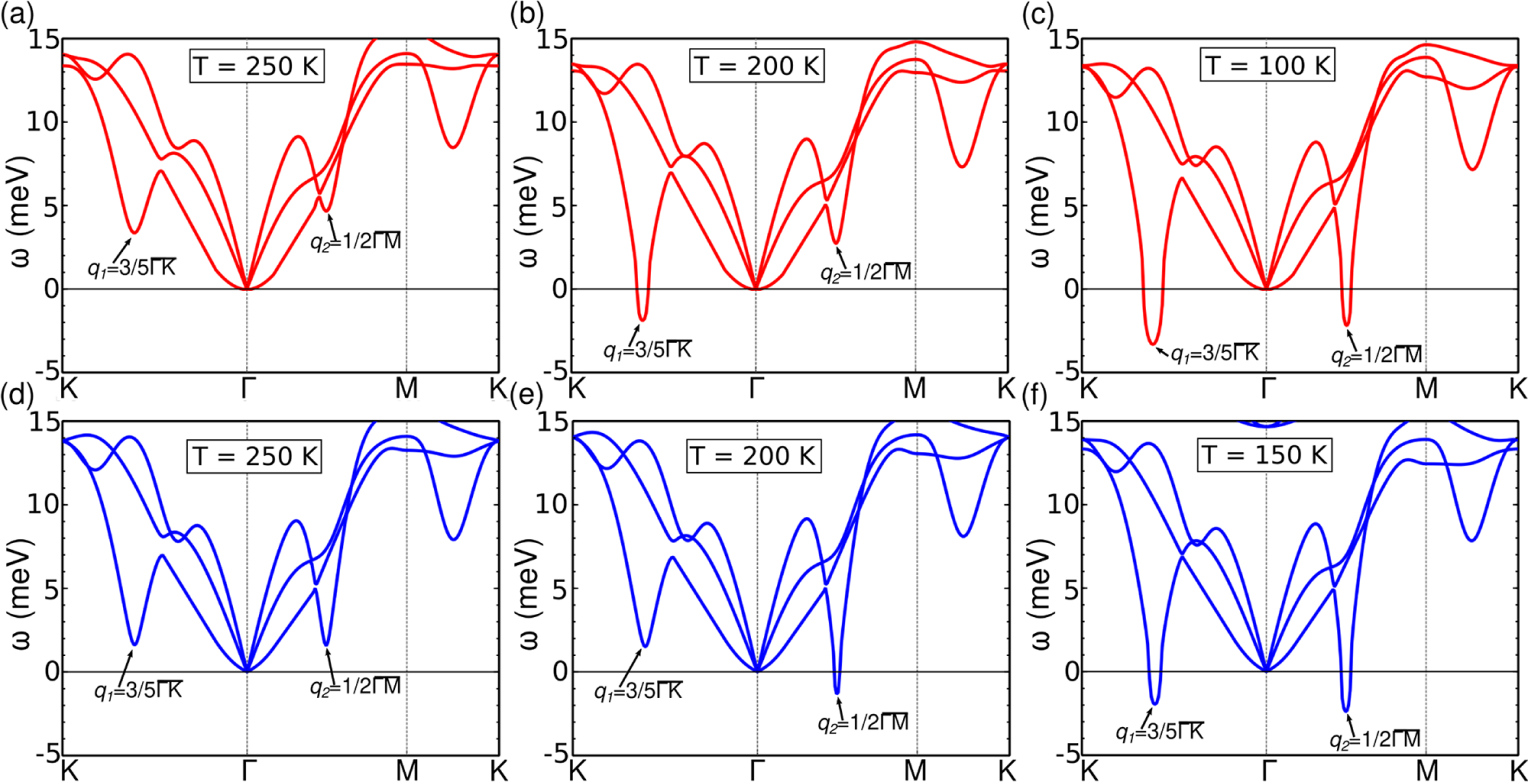
Nonperturbative anharmonic calculations of the NS of monolayer VSe_2_. (a–c) For lattice parameter *a* = 3.35 Å and temperatures of 250, 200, 100 K respectively. (d–f) For lattice parameter *a* = 3.30 Å and temperatures 250, 200, 150 K. The phonon melting occurs first at the *q*_1_(*q*_2_) point for *a* = 3.35 Å (*a* = 3.30 Å) as shown in panels (b) and (e).

To analyze in more detail the competition between the two CDW orders as a function of the lattice parameter, Figure 3a shows the temperature evolution of the frequency of the phonon mode that softens at *q*_1_ and *q*_2_. For the larger lattice parameter, *a* = 3.35 Å, the frequency at *q*_1_ becomes negative (imaginary) at higher temperature than at *q*_2_, and hence the  CDW order is the dominant (left panel in Figure 3a in red). The opposite behavior is observed for the small lattice parameter *a* = 3.30 Å. The frequency at *q*_2_ becomes negative at higher temperature than that at *q*_1_, and hence the 4 × 4 CDW order is dominant (right panel in Figure 3a in blue). From Figure 3a we can obtain the transition temperature for each lattice parameter: for *a* = 3.35 Å the  order emerges at *T*_CDW_ = 217 K, while for *a* = 3.30 Å the 4 × 4 order arises at *T*_CDW_ = 223 K. Importantly, our anharmonic calculations including the zero point energy^44^ predict an associated in-plane pressure of 0.7 GPa for *a* = 3.35 Å and 1.3 GPa for *a* = 3.30 Å. Considering that its in-plane pressure is lower, these results point out that the intrinsic CDW order in monolayer VSe_2_ is  with a *T*_CDW_ = 217 K, which is in perfect agreement with the experiments on refs (20) and (23) and that the 4 × 4 order, which is the in-plane projection of the bulk 4 × 4 × 3 CDW order, appears only under strain. Our results provide an explanation for the different CDW orders observed for small variations (∼1.5%) of the lattice parameter.^23,27^ Note that, eventually, other modulations could appear in monolayer VSe_2_ as experimentally reported.^25,26,36^ However, our results show that the  and 4 × 4 modulations are the intrinsic CDW orders and point out that those different modulations are a consequence of the interplay between the highly dynamically unstable NS of VSe_2_ monolayer and the particular substrate.

**Figure 3:**
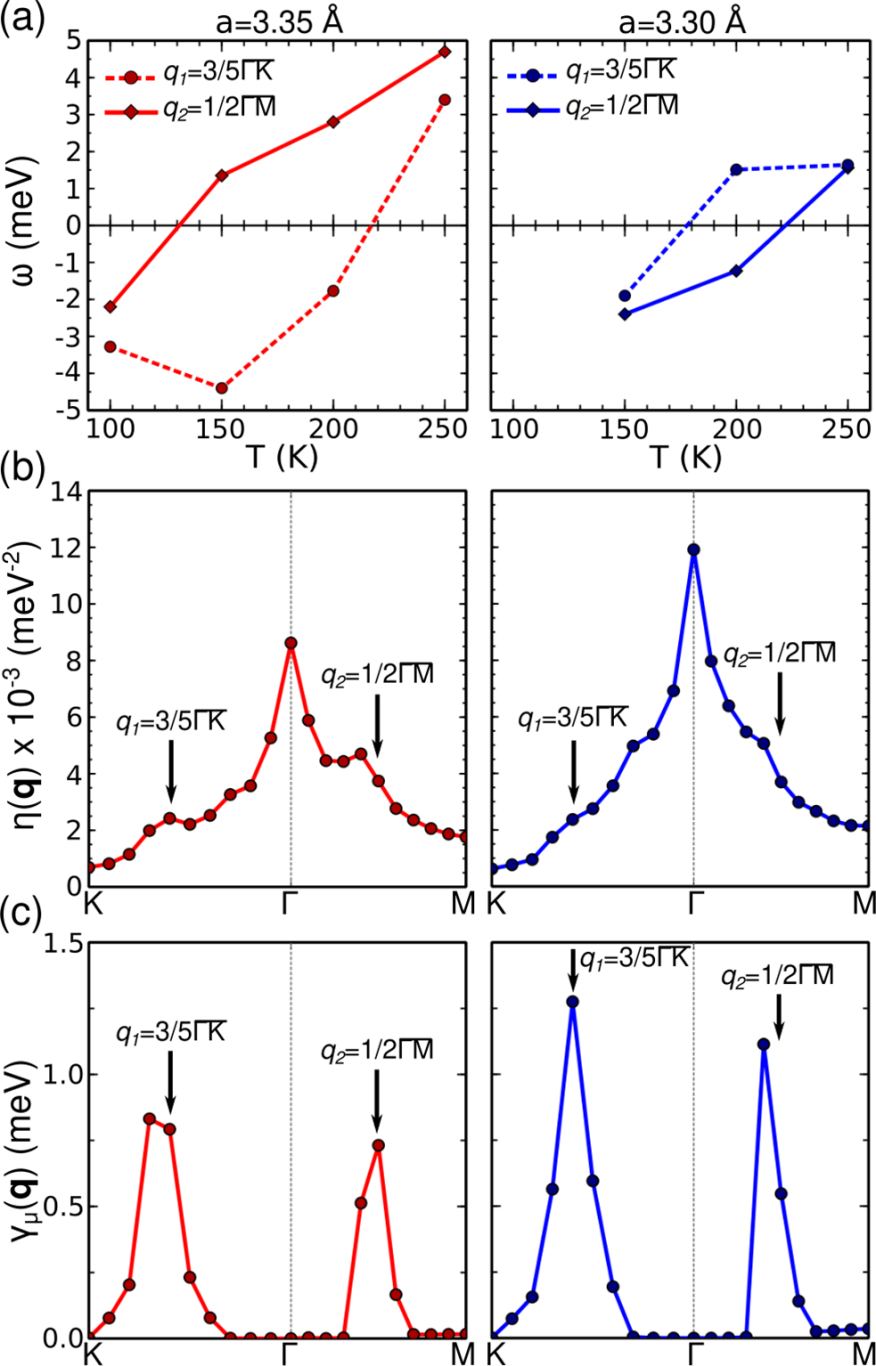
(a) Temperature evolution of the frequencies of the softened modes at *q*_1_ and *q*_2_ as obtained in the SSCHA calculations. (b) Nesting function η(*q*). It does not clearly peak at *q*_1_ and *q*_2_. (c) Phonon line width γ_μ_(**q**) given by the electron–phonon interaction. Sizable peaks appear at the CDW *q*-vectors. All of the results for *a* = 3.35 Å (*a* = 3.30 Å) are shown in red (blue) in the left (right) panels.

Having established the competition between the two intrinsic CDW orders of monolayer VSe_2_ as a function of the lattice parameter, we study now the origin of these CDW orders. In order to do so, we use DFPT to compute both the nesting function η(**q**) (Figure 3b), which is given by
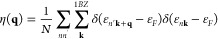
1and the phonon line width associated with the electron–phonon interaction (see Figure 3c).
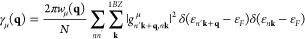
2

In eqs 1 and 2 ϵ_*n***k**_ is the energy of band *n* with wavenumber **k**, ϵ_*F*_ the Fermi energy, and *N* is the number of **k** points in the sum over the first Brillouin zone (1BZ). The nesting function peaks for **q** indicates that nested regions of the Fermi surface connect and, thus, reveal if the instability emerges from a purely electronic instability. The equation for γ_μ_(**q**) is very similar to the nesting function, but the value is weighted by the mode μ and momentum **q** dependent electron–phonon matrix elements  and, thus, reveals if the instability emerges from electron–phonon interactions. The electron–phonon line width is independent of the phonon frequency *w*_μ_(**q**) as the electron–phonon matrix elements scale as *w*_μ_(**q**)^−1/2^. It is worth noting that both quantities need to be computed in order to establish the origin of the CDW orders. For 2D systems like monolayer VSe_2_, despite the existence of nesting conditions at the **q** points associated with the CDW orders, a purely electronic picture does not suffice to produce a CDW order and electron–phonon interactions play a key role to drive the transition.^48^ Therefore, the direct comparison between the nesting function and the electron–phonon line width allows us to establish which is the main driving force of the CDW orders in monolayer VSe_2_.

We can see in Figure 3b that, for both lattice parameters the nesting function does not show any strong peak at the CDW vectors despite the existence of small shoulders near *q*_1_ and *q*_2_. In a different way, Figure 3c shows that the phonon line width coming from the electron–phonon interaction abruptly peaks at both *q*_1_ and *q*_2_ for both lattice parameters, meaning that in all cases the enhancement comes from the mode and momentum dependence of the electron–phonon matrix elements. Therefore, the two intrinsic CDW orders developed by monoloyer VSe_2_ are driven by the electron–phonon coupling, in agreement with the theoretical predictions for 2D systems.^48^ Besides, the electron–phonon interaction also plays a key role in the CDW transition in 3D systems, as in bulk 1*T*-VSe_2_, in which case the presence of nesting is symbolic.^30^

The analysis about the stability of the different CDW orders as a function of strain was performed with a nonlocal van der Waals density exchange-correlation functional.^49^ The reason for this is that this functional allows us to properly describe both bulk and monolayer limits of VSe_2_, oppositely to the widely used GGA-PBE functional (see the Supporting Information for a more detailed description of the election of the exchange correlation functional to study the CDW orders of VSe_2_). In the latter case, a huge overestimation of *T*_CDW_ occurs in bulk due to the lack of van der Waals interactions that cause a melting of the CDW phase.^30^ Motivated by these results in the bulk, we explore here the effect that external van der Waals interactions may cause in the CDW orders of monolayer VSe_2_. These van der Waals interactions might naturally appear by proximity effect between the analyzed monolayer and other layers, such as the substrate or in van der Waals heterostructures. We will introduce these external interactions in a phenomenological way. This approach allows us to derive general conclusions for the pure effect of van der Waals interactions on CDW orders, i.e., factoring out effects that could appear in particular substrates, such as charge transfer.^40^

First, to illustrate the effect of external van der Waals forces on the CDW, we make use of a simple one-dimensional double-well fourth order potential
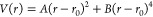
3where *A* and *B* are the coefficients of the different powers, *r* is the position of the atoms, and *r*_0_ is the equilibrium atomic position in the high temperature phase. The CDW occurs when the free energy calculated with this potential is lower at *r* – *r*_0_ ≠ 0 than at *r* – *r*_0_ = 0. Obviously the lower and wider the minimum of the well the more probable it is to find a broken-symmetry CDW order. We can now add to the potential *V*(*r*) a *E*_LJ_(*r*) Lennard-Jones energetic contribution to mimic the role played by external van der Waals interactions
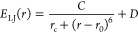
4where *C* is the coefficient that controls the strength of the Lennard-Jones interactions, *r*_*c*_ is a cutoff radius that prevents a divergence at *r* = *r*_0_, and *D* = *C*/*r*_*c*_ is simply a constant that fixes the potential to be equal to 0 at *r*_0_. The effect of the Lennard-Jones interactions on *V*(*r*) can be seen in Figure 4a for different values of *C*. We can observe that an increase in the strength of the Lennard-Jones interactions makes the potential shallower. Therefore, Lennard-Jones interactions tend to quench the low-temperature CDW phase and promote the high-temperature symmetric phase.

**Figure 4:**
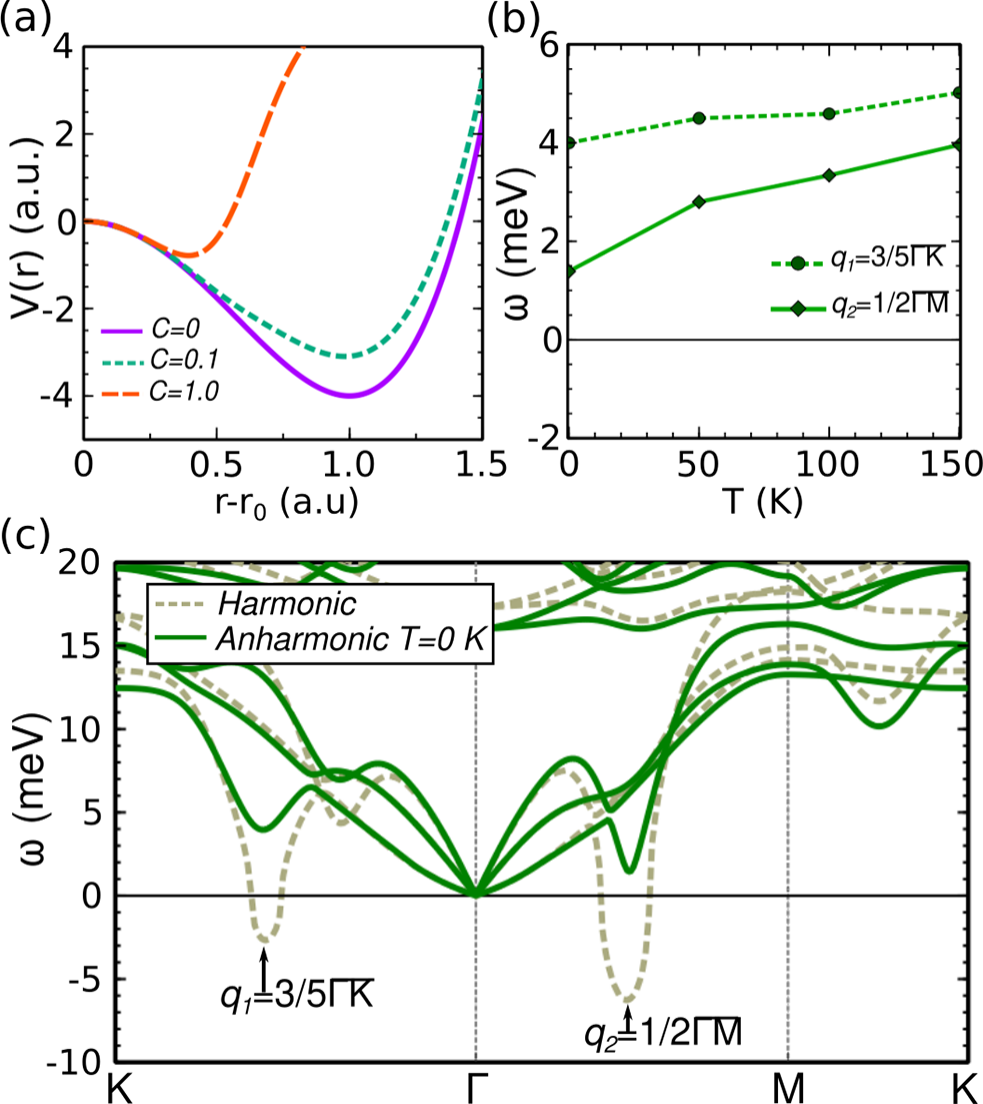
(a) *V*(*r*) potential as a function of the atomic position *r* described by eqs 3 and 4 in arbitrary units (a.u.) for different *C* values. Particular values of *A* = −8, *B* = −*A*/2, and *r*_*c*_ = 0.1 are considered. (b) Temperature evolution of the soften modes’ frequencies at *q*_1_ and *q*_2_ when anharmonicity and Lennard-Jones interactions are included. (c) Harmonic and anharmonic phonon band structures at *T* = 0 K for monolayer VSe_2_ including Lennard-Jones contributions.

We can confirm this simple picture in the particular case of monolayer VSe_2_ by including an energy term like the one shown in eq 4 through the Grimme’s semiempirical approach in our SSCHA calculations on top of the PBE functional.^50^Figure 4c shows the harmonic phonon band structure including energy contributions from Lennard-Jones interactions. (These calculations are for *a* = 3.35 Å, but qualitatively the results hold for any other lattice parameter. The strength of the Lennard-Jones interactions was set to the one considered by default by the Grimme’s semiempirical correction implemented in the QUANTUM ESPRESSO package^51,52^) The instability at *q*_1_ and *q*_2_ is reduced compared to the harmonic bands without the Lennard-Jones contribution (see the Supporting Information for a better visualization of this effect at the harmonic level.). In this situation, anharmonic effects are able to suppress both CDW orders by stabilizing the softened phonons even at 0 K, as shown in Figure 4(c). This effect can be also analyzed in Figure 4(b), where the evolution of the frequencies of the softened modes at *q*_1_ and *q*_2_ as a function of temperature is shown. The frequencies remain stable at any temperature. Therefore, this demonstrates that the combination of Lennard-Jones interactions and anharmonicity can destroy the CDW orders, stabilizing the NS phase at low temperatures. Note that here we have considered a strength of the Lennard-Jones interactions that quenches both CDW orders. However, this strength could be modulated by the parameter *C* in eq 4, providing simply a decrease of *T*_CDW_, but not a suppression of the CDW order, as reported for bulk.^30^ This effect may also be related with the enhancement of the CDW order in the 2D limit,^34^ in which this kind of interactions decreases. Therefore, not only strain but also Lennard-Jones interactions can explain the huge variability of transition temperatures and CDW orders reported in experiments where VSe_2_ is grown on different substrates.^23,25−27^

Finally, note that monolayer VSe_2_ has attracted great attention due to its proximity to an itinerant ferromagnetic state, which is suppressed by the presence of CDW orders.^19,20^ Our analysis suggests that a ferromagnetic state in monolayer VSe_2_ may be possible if its intrinsic CDW orders are suppressed by external Lennard-Jones interactions. This tuning could be implemented by substrate engineering or by artificial design of van der Waals heterostructures. In particular, combining compounds that display CDW orders with ferroelectric materials, which provide strong dipolar interactions, might allow an electric control of CDW phases and the emergence of other competing orders. This mechanism to tune or destroy CDW orders is generic and could be extended to other similar systems, offering a novel platform to engineering new functional materials.

In conclusion, to solve previous experimental contradictions found for the CDW orders of monolayer VSe_2_, in this work we analyze the CDW orders of monolayer VSe_2_ using nonperturbative anharmonic phonon calculations that allow us to determine the CDW orders of this system and their corresponding transition temperatures. We analyze the effect of strain and external van der Waals interactions, since these two parameters are intrinsic to any experiment. We demonstrate the competition between two intrinsic CDW orders as a function of the lattice parameter. Variations of 1.5% in the lattice parameter are enough to drive the system from the  to the 4 × 4 order. Transition temperatures on the order of 220 K have been found for both CDW orders, in very good agreement with experiments. We show that external Lennard-Jones interactions tend to weaken or even suppress the CDW orders. These results together help to understand the great variability of CDW orders and associated *T*_CDW_’s found in the experiments of monolayer VSe_2_. Moreover, they pave the way to tune CDW orders occurring in van der Waals materials, thus promoting competing orders that might arise in these systems.

## Supporting Information Available

Detailed explanation of the computational methods, the election of the exchange correlation functional and the effect of external Lennard-Jones interactions at the harmonic level. The Supporting Information is available free of charge at https://pubs.acs.org/doi/10.1021/acs.nanolett.2c04584.

Detailed explanation of the computational methods, the election of the exchange correlation functional and the effect of external Lennard-Jones interactions at the harmonic level (PDF)
