# Supplementary material for: Anharmonicity Reveals the Tunability of the Charge Density Wave Orders in Monolayer VSe2
Source: Nano Lett. 2023 Feb 24;23(5):1794–800. doi: 10.1021/acs.nanolett.2c04584 (PMC9999451; doi:10.1021/acs.nanolett.2c04584)
Supplement: Supplementary file 1 — nl2c04584_si_001.pdf [file nl2c04584_si_001.pdf]

Supplemental Material: Anharmonicity reveals the tunability of the charge density wave orders in monolayer VSe_2

Adolfo Otero Fumega,[*,†] Josu Diego,[‡,¶] Victor Pardo,[§,‖] Santiago Blanco-Canosa,[⊥,#] and Ion Errea[*,‡,¶,⊥]

†*Department of Applied Physics, Aalto University, 02150 Espoo, Finland*

‡*Fisika Aplikatua Saila, Gipuzkoako Ingeniaritza Eskola, University of the Basque Country (UPV/EHU), 20018, San Sebastián, Spain*

¶*Centro de Física de Materiales (CSIC-UPV/EHU), 20018, San Sebastián, Spain*

§*Departamento de Física Aplicada, Universidade de Santiago de Compostela, 15782, Santiago de Compostela, Spain*

‖*Instituto de Materiais iMATUS, Universidade de Santiago de Compostela, 15782, Santiago de Compostela, Spain*

⊥*Donostia International Physics Center (DIPC), 20018, San Sebastián, Spain*

#*IKERBASQUE, Basque Foundation for Science, 48013 Bilbao, Spain*

E-mail: adolfo.oterofumega@aalto.fi; ion.errea@ehu.eus

Computational methods

Density Functional Perturbation Theory (DFPT) calculations

Harmonic phonon frequencies and electron-phonon matrix elements were calculated within density functional perturbation theory (DFPT)[1] as implemented in the Quantum ESPRESSO package.[2,3] The force calculations needed for DFPT calculations were performed making use of a non-local van der Waals exchange-correlation functional.[4,5] We used an ultrasoft pseudopotential that includes $4s^2$ $3d^3$ valence electrons for V and a norm-conserving one with $4s^2$ $4p^4$ electrons in the valence for Se. We used a plane-wave energy cutoff of 50 Ry for the wavefunctions and 550 Ry for the charge density. The Brillouin zone integrals were performed in a 32×32×1 k-point grid with a Methfessel-Paxton smearing[6] of 0.01 Ry. Harmonic phonon calculations were carried out in a 8×8×1 q-point grid. The nesting function and the electron-phonon linewidth were calculated using a 48×48×1 k-point grid and a Gaussian broadening of 0.003 Ry for the Dirac deltas.

The stochastic self-consistent harmonic approximation (SSCHA)

The stochastic self-consistent harmonic approximation (SSCHA)[7–10] is a quantum variational method on the free energy fully accounting for anharmonic effects at any temperature. The variational minimization is carried out with respect to a trial harmonic density matrix $\rho_{\mathcal{H}}$ that contains two groups of parameters: the force-constants $\mathbf{\Phi}$ and the *centroid* positions $\boldsymbol{\mathcal{R}}$. The centroid positions at the free energy minimum $\boldsymbol{\mathcal{R}}_{eq}$ are the average ionic equilibrium positions fully accounting for quantum, thermal and anharmonic effects. The temperature-dependent anharmonic phonon spectra at the static level are obtained from the diagonalization of a free energy Hessian based dynamical matrix, $D^{(F)}_{ab} = \frac{1}{\sqrt{M_a M_b}} \frac{\partial^2 F}{\partial \mathcal{R}_a \partial \mathcal{R}_b}\Big|_{\mathcal{R}_{eq}}$.[8] Following Landau's theory,[11] these phonons are enough to identify second-order structural phase transitions, being the transition critical temperature the one in which a phonon mode goes to null frequency.

The variational free energy minimization within the SSCHA method was performed by calculating forces on 4×4×1 supercells making use of DFT as implemented in Quantum ESPRESSO. We used the same non-local van der Waals exchange-correlation functional, pseudopotentials and parameters described in the previous section, but with a 4×4×1 grid in the unit cell for the Brillouin zone integrals. The theoretical anharmonic phonon spectra shown in the paper were calculated in the static limit of the SSCHA theory; based on the free energy Hessian formalism. The difference between anharmonic and harmonic dynamical matrices was interpolated to a finer grid of size 8×8×1 in order to obtain other anharmonic phonon frequencies in more **q** points.

Election of the exchange correlation functional

Importantly, in the anharmonic analyses carried out in this manuscript with the SSCHA methodology, we have considered the a non-local van der Waals exchange-correlation functional.[5] In this section we justify such a choice.

As reported in Ref.,[12] it is fundamental to include van der Waals interactions to provide a good description of the transition temperature from the NS to the CDW state. Figure 1a shows a summary of the results for bulk VSe_2 obtained in Ref.[12] using the GGA-PBE exchange-correlation functional[13] and including the van der Waals interactions in a semiempirical way.[14] Moreover, we have included our calculations using the non-local van der Waals exchange-correlation functional.[5] We can observe that the prediction using GGA-PBE is totally wrong since it does not show any transition from the CDW to the NS state at temperatures below 300 K, being T_{CDW} = 110 K. The reason for this result is that van der Waals interactions are not taken into account at the PBE level. In contrast, we can observe that for the semiempirical approach and for the non-local van der Waals functional the phase transition is captured below 200 K. In particular, the non-local van der Waals functional predicts a transition temperature of T_{CDW} = 190 K, this overestimation might be due to

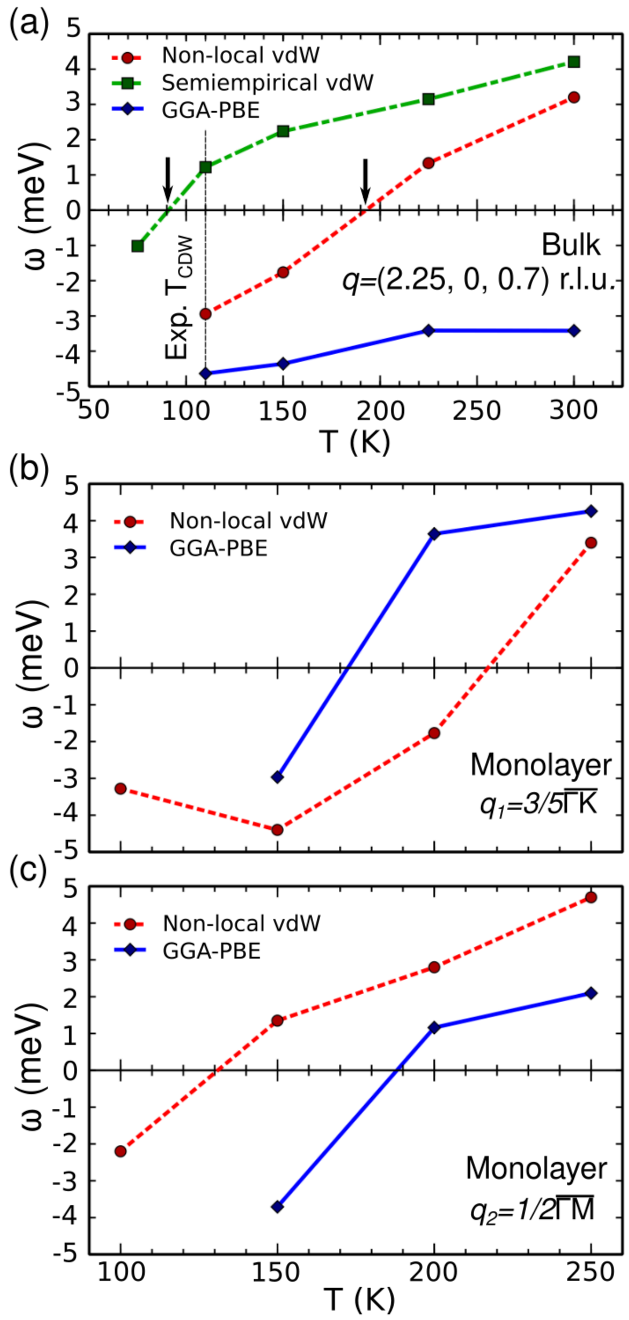

Figure 1: Temperature evolution of the frequencies of the softened modes associated to the CDW orders for different exchange correlation functionals: (a) Evolution for bulk VSe_2 at q =(2.25,0.0,0.7), data for the semiempirical van der Waals and the GGA-PBE functional taken from Ref.[12] The predicted transition temperatures for each functional are highlighted with an arrow, the experimental T_{CDW} is also indicated. No transition to the NS is observed below 300K for the GGA-PBE functional. (b,c) Evolution for monolayer VSe_2 at $q_1 = 3/5\overline{\Gamma K}$ and $q_2 = 1/2\overline{\Gamma M}$ associated to the $\sqrt{3} \times \sqrt{7}$ and 4×4 modulations respectively considering $a = 3.35$ Å.

the lack of some non-local effects that are difficult to capture in a DFT exchange-correlation functional. The semiempirical approach is based on Lennard-Jones terms that are added in a non-*ab initio* way. For the default values set in the QUANTUM ESPRESSO package for the strength of this interaction $T_{CDW} = 90$ K, thus producing a small underestimation of the transition temperature. Of course in this semiempirical approach the strengh of the van der Waals interactions could be varied to describe better the transition, however note that this would depart from an *ab initio* determination of the T_{CDW}.

Figures 1b and 1c show the temperature evolution of the frequencies of the softened modes at $q_1 = 3/5\overline{\Gamma\text{K}}$ and $q_2 = 1/2\overline{\Gamma\text{M}}$ associated to the CDW orders found in monolayer VSe_2. Results for the GGA-PBE and the non-local exchange correlation functional are shown using an in-plane lattice parameter $a = 3.35$ Å in the calculations. We can see in these figures the predicted dominant CDW order and its corresponding T_{CDW}. In the monolayer limit, one might expect that van der Waals interactions are going to be highly suppressed and hence it is going to be irrelevant whether considering the PBE or the non-local approximation. However, there are a couple of important reasons to select the non-local van der Waals functional in the monolayer. First, as we have seen, the non-local functional is able to describe both the bulk and the monolayer limit. This allows us to drive conclusions about the trend of the CDW order as a function of the dimensionality. Our calculations using non-local van der Waals predict an enhancement of the CDW order in the monolayer limit of around ∼20-30 K, in good agreement with experimental results.[15] Second, we can observe from Figs. 1b and 1c that the PBE functional predicts the 4×4 CDW order to be the dominant one at $a = 3.35$ Å, while the non-local functional predicts the $\sqrt{3} \times \sqrt{7}$ to be the dominant one. This disagreement between both functionals stems from the fact that the polarization vectors associated to the CDW orders have out-of-plane components (see Figs. 1c and 1d of the main text) that may induce non-local effects in the monolayer. Taking into account all this, plus the fact that monolayer VSe_2 presents a highly dynamically-unstable NS justifies the choice of the non-local van der Waals functional as the best choice to determine

the competition between both CDW orders. Indeed, we found that the predictions of this functional are in agreement with the reported experimental results.[16,17] Finally, notice that using the semiempirical approach to drive conclusions about the intrinsic CDW orders and their competition in the monolayer is not an option, since this treatment is not *ab initio* and the intrinsic non-local effects that might arise in the monolayer cannot be anticipated. However, it can be used to study the qualitative effect of adding external van der Waals interaction to the monolayer, for instance by proximity effects of the monolayer with a substrate or in a van der Waals heterostructure, as we have performed in the main text when considering the effect of external Lennard-Jones energy terms entering in the monolayer.

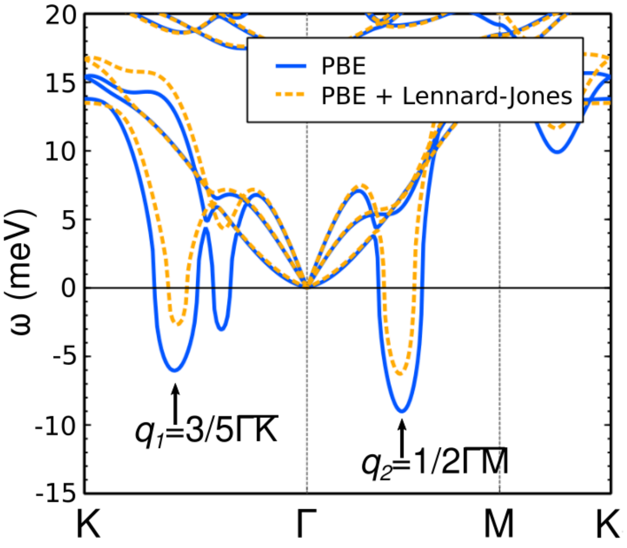

Figure 2: Harmonic phonon calculations for monolayer VSe_2. We can observe that including external Lennard-Jones interactions decreases the instability of the unstable modes associated with the CDW orders at q_1 and q_2.

Effect of external Lennard-Jones interactions at the harmonic level

The effect of adding external Lennard-Jones interactions in monolayer VSe_2 can be seen in Fig. 2. We can observe that, at the harmonic level, Lennard-Jones interactions reduce

the instabilities associated to the CDW orders. As explained in the main text, this is the expected behavior, since this kind of energy terms lead to the renormalization of the potential energy surface, approaching the system to the high-temperature, symmetric, normal state phase.

References

(1) Baroni, S.; de Gironcoli, S.; Dal Corso, A.; Giannozzi, P. Phonons and related crystal properties from density-functional perturbation theory. *Rev. Mod. Phys.* **2001**, *73*, 515–562.

(2) Giannozzi *et al.*, P. QUANTUM ESPRESSO: a modular and open-source software project for quantum simulations of materials. *J. Phys. Condens. Matter* **2009**, *21*, 395502.

(3) Giannozzi *et al.*, P. Advanced capabilities for materials modelling with Q uantum ESPRESSO. *Journal of Physics: Condensed Matter* **2017**, *29*, 465901.

(4) Hohenberg, P.; Kohn, W. Inhomogeneous Electron Gas. *Phys. Rev.* **1964**, *136*, B864–B871.

(5) Thonhauser, T.; Cooper, V. R.; Li, S.; Puzder, A.; Hyldgaard, P.; Langreth, D. C. Van der Waals density functional: Self-consistent potential and the nature of the van der Waals bond. *Phys. Rev. B* **2007**, *76*, 125112.

(6) Methfessel, M.; Paxton, A. T. High-precision sampling for Brillouin-zone integration in metals. *Phys. Rev. B* **1989**, *40*, 3616–3621.

(7) Errea, I.; Calandra, M.; Mauri, F. Anharmonic free energies and phonon dispersions from the stochastic self-consistent harmonic approximation: Application to platinum and palladium hydrides. *Phys. Rev. B* **2014**, *89*, 064302.

(8) Bianco, R.; Errea, I.; Paulatto, L.; Calandra, M.; Mauri, F. Second-order structural phase transitions, free energy curvature, and temperature-dependent anharmonic phonons in the self-consistent harmonic approximation: Theory and stochastic implementation. *Phys. Rev. B* **2017**, *96*, 014111.

(9) Monacelli, L.; Errea, I.; Calandra, M.; Mauri, F. Pressure and stress tensor of complex anharmonic crystals within the stochastic self-consistent harmonic approximation. *Phys. Rev. B* **2018**, *98*, 024106.

(10) Monacelli, L.; Bianco, R.; Cherubini, M.; Calandra, M.; Errea, I.; Mauri, F. The stochastic self-consistent harmonic approximation: calculating vibrational properties of materials with full quantum and anharmonic effects. *Journal of Physics: Condensed Matter* **2021**, *33*, 363001.

(11) Landau, L. D.; Lifshitz, E. M. *Fluid Mechanics, Second Edition: Volume 6 (Course of Theoretical Physics)*, 2nd ed.; Course of theoretical physics / by L. D. Landau and E. M. Lifshitz, Vol. 6; Butterworth-Heinemann, 1987.

(12) Diego, J.; Said, A. H.; Mahatha, S. K.; Bianco, R.; Monacelli, L.; Calandra, M.; Mauri, F.; Rossnagel, K.; Errea, I.; Blanco-Canosa, S. van der Waals driven anharmonic melting of the 3D charge density wave in VSe2. *Nature Communications* **2021**, *12*, 598.

(13) Perdew, J. P.; Burke, K.; Wang, Y. Generalized gradient approximation for the exchange-correlation hole of a many-electron system. *Phys. Rev. B* **1996**, *54*, 16533–16539.

(14) Grimme, S. Semiempirical GGA-type density functional constructed with a long-range dispersion correction. *Journal of Computational Chemistry* **2006**, *27*, 1787–1799.

(15) Pásztor, Á.; Scarfato, A.; Barreteau, C.; Giannini, E.; Renner, C. Dimensional crossover

of the charge density wave transition in thin exfoliated VSe 2. *2D Materials* **2017**, *4*, 041005.

(16) Feng, J. et al. Electronic Structure and Enhanced Charge-Density Wave Order of Monolayer VSe2. *Nano Letters* **2018**, *18*, 4493–4499, PMID: 29912565.

(17) Chen, P.; Pai, W. W.; Chan, Y.-H.; Madhavan, V.; Chou, M. Y.; Mo, S.-K.; Fedorov, A.-V.; Chiang, T.-C. Unique Gap Structure and Symmetry of the Charge Density Wave in Single-Layer VSe_2. *Phys. Rev. Lett.* **2018**, *121*, 196402.
